# Controversies in the management of intra-articular fractures of distal humerus in adults

**DOI:** 10.4103/0019-5413.80039

**Published:** 2011

**Authors:** Sudhir Babhulkar, Sushrut Babhulkar

**Affiliations:** Department of Orthopedics, Indira Gandhi Medical College, Nagpur, India; 1 Sushrut Hospital, Reseach Center and Postgraduate Institute of Orthopedics, Nagpur, India

**Keywords:** Distal humerus, intra-articular fracture, olecranon osteotomy, plate fixation, tension band wire

## Abstract

**Background:**

The surgical approach, type of olecranon osteotomy, method of stabilization of osteotomy, type of fracture stabilization, orthogonal vs parallel plate fixation, need for transposition of ulnar nerve, place for primary total elbow replacement, and type of rehabilitation schedule after surgical fracture treatment are the controversial issues in the treatment of complex intra-articular distal humerus fractures (C2 and C3) in adults. Severe comminution, bone loss, and osteoporosis at the site of distal articular fractures of humerus often lead to unsatisfactory results due to inadequate fixation. We hereby report the outcome of a series of intracondylar fractures of the humerus treated by open reduction and internal fixation and discuss the controversies in light of published literature.

**Materials and Methods:**

One hundred and eighty-four patients of intra-articular fractures of distal humerus (C2 and C3) were operated by posterior transolecranon approach between January 1980 and December 2008. Initially, in the first part Chevron intra-articular osteotomy (*n*=108) was performed out of which 94 have been published in another publication. In later second part (1993 onward), extra-articular olecranon osteotomy (*n*=76) was routinely performed. Both columns were stably fixed by orthogonal methods; (n=174) however, during the last 2 years, in 10 patients with severe comminution with bone loss, stabilization was achieved by parallel plating. The osteotomy was routinely stabilized by tension band wiring with two parallel K-wires introduced up to the anterior ulnar cortex. The results were evaluated by the staging system of Caja *et al*. at a minimum follow-up of 2 years.

**Results:**

In the first part of the study (*n*=94), there was delayed union in 4% (*n*=4), with the fracture taking more than 20 weeks for union. There was delayed union of ulnar osteotomy (n=3) and failure of one tension band wiring, requiring revision. Some loss of motion was seen in 20% of cases and these patients did not achieve full flexion and extension. However, all these patients had useful range of function, with 20°–110° of flexion and full pronation-supination. As per the staging system of Caja *et al*., the results were in the range of excellent to good in 72% cases (*n*=67), fair in 19% (*n*=18), and poor in 9% patients (*n*=9). In the second part of study (*n*=90) dual plate fixation of both columns by orthogonal methods (n=80) and parallel plate fixation in 10 patients was performed. The results were excellent to good in 78 patients (86%).

**Conclusions:**

The high rate of union can be achieved in complex intra-articular fractures of distal humerus if the proper principles of stable fracture fixation are followed, i.e., a posterior transolecranon approach and dual fixation of both columns and restoration of the continuity of articular surface. The stability achieved by this technique permits institution of early intensive physiotherapy to restore elbow function.

## INTRODUCTION

Intra-articular fractures of the distal humerus constitute 0.5%–7% of all fractures and 30% of elbow fractures.^1^ Distal humeral fracture occurs in the younger age-groups secondary to high-energy trauma and in elderly women as a result of relatively low-energy trauma.^1^ The chances of functional impairment and deformity are very high following conservative treatment of such distal intra-articular fractures of the humerus, and stable internal fixation may be difficult to achieve due to the complexity of the fracture and associated osteoporosis.^2^ Good anatomical alignment, stabilization, and early mobilization can provide satisfactory results. Severe comminution, bone loss, and osteopenia predispose to unsatisfactory results because of inadequate fixation of the fracture.^2^,^3^ Over 25% of such fractures develop significant complications during treatment and a few of them may need further surgery.^4^ Standard surgical techniques are used for fixation of both columns, using a combination of reconstruction plates, dynamic compression plates, locking compression plates, and screws and K-wires. In rare situations primary total elbow arthroplasty (TEA) may be considered.

However, in the management of intra-articular distal humerus fractures in adults controversy still exists regarding the surgical approach, type of olecranon osteotomy, method of stabilization of osteotomy, type of fracture stabilization, use of orthogonal or parallel plate fixation, need for transposition of ulnar nerve, place for primary TEA, and type of rehabilitation schedule after surgical fracture treatment. In this article we report the outcome of a series of intracondylar fractures of the humerus treated by open reduction and internal fixation (ORIF) and discuss the controversies in light of available evidence in literature.

## MATERIALS AND METHODS

This is a retrospective analysis of intra-articular fractures (184 patients) of distal humerus (C2 and C3) treated by us over a period of 28 years [Table 1]. 197 patients were operated 13 patients did not report for follow-up and were not contactable and hence were not included in the present analysis. All patients had recent injuries and reported within 1 to 15 days of injury. All patients were operated by the posterior transolecranon approach after olecranon osteotomy. The study comprises two parts: part I, included 94 patients operated during 1980–1992 by Chevron intra-articular osteotomy, with stabilization by tension band wiring. Both columns were stably fixed routinely by orthogonal plate application (90-90). The details were published in an article in 1995.^5^ The second part of study (1993–2008) comprises 90 patients operated by a similar posterior transolecranon approach, with extrarticular osteotomy (n=76) and Chevron osteotomy (n=14) in the early period, which was stabilized by tension band wiring. On 80 occasions both columns were stabilized by orthogonal fixation, using plates on both sides; however, in the last 2 years, in 10 patients with severe comminution and bone loss, stabilization was achieved by locking compression parallel plating.

**Table 1:** Age, sex, side involved, type of fracture pattern

Age-group (years)	No. of patients	Sex		Fracture type		Side involved	
		Male	Female	C2	C3	Right	Left
Part I							
20–30	32	24	8	21	11	25	7
30–40	43	34	9	28	15	27	16
40–50	19	15	4	12	7	13	6
Total	94	73	21	61	33	65	29
Part II							
20–30	26	22	4	18	8	20	6
30–40	39	28	11	27	12	30	9
40–50	25	16	9	15	10	19	6
Total	90	66	24	60	30	69	21
Total of parts I and II	184	139	45	121	63	134	50

Following surgical stabilization, the suction drain was removed after 48 h. The posterior plaster slab was retained for 5–7 days. Gentle elbow mobilization was started thereafter and the patient was discharged with advice to attend daily active and assisted physiotherapy. Patient were reviewed every 3 weeks for the first 2 months, every month for next 6 months, and then every 6 months for a minimum of 2–3 years. Patients were assessed using the scoring system of Caja *et al*. every 2–3 months.^6^

## RESULTS

The scoring system of Caja *et al*.^6^ takes into consideration the four parameters of pain, range of motion, radiological quality of surgical reduction, and the level of activity. Pain was absent in more than 60% of the patients (*n*=110) 4–6 weeks after surgery, with occasional pain being reported by 20% (*n*=36), especially after daily heavy activity. Range of motion was better in younger subjects (between 20–40 years) than in the older age-groups. The level of activity was also good in the younger age-groups, with better painless elbow function. An excellent result is considered a stable, pain-free elbow, with a nearly normal range of motion of ≥120°.

In first part of the study, there was delayed union of humerus fracture in 4% of cases (n=4), requiring more than 20 weeks for union; delayed union of ulnar osteotomy was seen on three occasions.^5^ There was also failure of one tension band wiring, requiring revision. The patients did not achieve full flexion and extension in 19 cases (20%). However, all these patients had useful range of motion, with 20°–110° of flexion and full pronation-supination. There was 2% (*n*=2) incidence of postoperative ulnar neuritis, required neurolysis after 10–12 weeks for intractable tingling and numbness. The results were excellent to good in 72% (n=67) cases, fair in 19% (n=18), and poor in 9% (n=9) as per the staging system of Caja *et al*.^6^ In the second part of the study comprising 90 patients (with dual plate fixation of both columns by orthogonal methods in 80 patients and with parallel plate fixation in 10 patients) the results were excellent to good in 78 patients (86%). In 9 patients (10%) the results were fair, with little limitation in the useful range of movements. The results were poor in three patients (4%) and these patients required further surgery for achieving union; one patient required removal of the painful tension band wiring.

The demographic profile of both parts of studies had similar fracture profile and the results were not statistically significantly different.

## DISCUSSION

The ORIF remains the standard of care in the treatment of intra-articular distal humerus fractures in the physiologically active patient.^5^,^7^,^8^ The treatment of distal humeral fractures is labor-intensive and complex and, expectedly, there is a high incidence of complications.^9^ Severe comminution, bone loss, and osteopenia predispose distal humeral fractures to unsatisfactory results due to inadequate fixation.^2^,^4^

The surgical stabilization should be undertaken as planned surgery with careful preoperative planning. Using good traction films (anteroposterior and lateral views), tracings are made, carefully noting the fracture lines and outlining major fracture fragments; this should be compared to the tracings of the intact uninjured distal humerus of the opposite side.^10^ The surgical tactics can be practiced on this paper tracing, where the perfect anatomy of the distal humerus is reconstructed. The appropriate implants are then traced in position while planning the size and location of plates and the position and number of screws.

### Position of the patient

**Figure 1:**
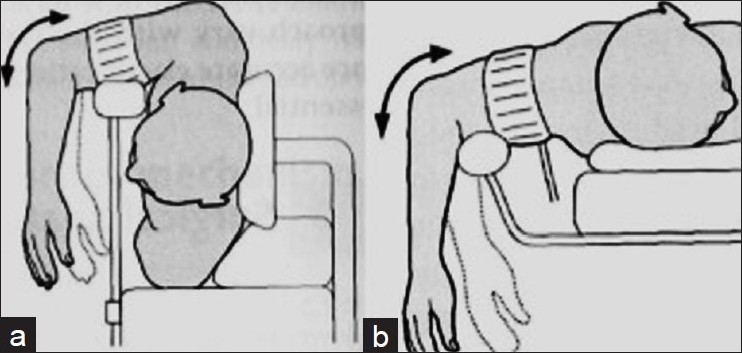
(a) The lateral decubitus position for fracture exposure (b) The prone position for fracture exposure

The position of patient during surgery varies according to the complexity of the fracture. The patient may be positioned in the lateral decubitus position on a bolster or beanbag, with the entire upper extremity draped free [Figure 1].^5^,^10^–^14^ The advantages of this position include ease of access to the posterior elbow for fracture fixation, without the need for extra assistants. The patient can also be positioned prone with the arm at 90° abduction and the elbow at 90° flexion over a radiolucent arm board [Figure 1].^10^ Care should be taken to pad and protect all bony prominences. When the patient cannot be placed in the prone or lateral position, it can be operated in the supine position with a small stack of towels placed underneath the ipsilateral scapula and with the arm draped across the chest after sterile preparation.^14^–^16^

Patients are usually operated under brachial block and upper-arm pneumatic tourniquet, with routine deflation after 2 h for procedures that exceed this length of time. The use of a sterile tourniqu*et al*lows for ease of removal if more proximal exposure of the humerus is needed.^10^,^17^,^18^ The posterior iliac crest should also be prepared and draped for procuring bone graft in patients with bone loss at the fracture site.

Adequate exposure is critical for the visualization of the fracture fragments during open reduction and fixation, and it is generally agreed that the best exposure of both columns of the distal part of the humerus and the articular surface is achieved by osteotomy through a posterior transolecranon approach.^5^,^11^,^12^,^19^–^24^ In 1969 the transolecranon approach was popularized by Cassebaum^19^ though MacAusland had described the technique as early as in 1915. There are several modifications to this technique, such as the chevron-shaped osteotomy, commonly advocated by the AO (Arbeitsgemeinschaft fur osteosynthesefragen) group.^21^ The chevron osteotomy increases rotational and translational stability at the time of surgery and increases the contact area for achieving the bony union. Since 1993 we are performing extrarticular olecranon osteotomy routinely as advocated by Muller *et al*.^21^ It has the advantage of same exposure of articular surface of distal humerus without disturbing the articular surface of olecranon, thereby reducing the chances of elbow stiffness and improving the range of movements postoperatively. To avoid complications, it is strongly advisable that the osteotomy should be fixed by tension band wiring with two tightening loops.^5^,^11^,^12^,^24^–^26^ Alternatively, a precontoured olecranon plate may be used.

Significant complications are associated with the osteotomy; for example, delayed union, nonunion, and symptomatic olecranon fixation. To eliminate these complications, operative exposures that involve mobilization of the extensor mechanism from the proximal ulna and/or distal humerus have been advocated.^10^,^13^,^14^,^17^,^27^ However, these alternative exposures do not offer the same degree of articular visualization as the olecranon process remains intact, partially obstructing the distal humeral trochlea. Specifically, Wilkinson and Stanley^22^ have demonstrated in a cadaver model study that the proportion of articular surface exposed in the triceps-splitting, triceps-reflecting, and olecranon-osteotomy approaches was 35%, 46%, and 57%, respectively. Furthermore, triceps-elevating exposures can be associated with weakness of extension or even rupture of the triceps, although a recent study of a central triceps peel showed similar extension strength as in patients undergoing olecranon osteotomy.^7^

### Surgical approach

Generally, intra-articular fractures of the distal humerus are accessed by the posterior approach, which gives excellent exposure of the articular fragments of the distal humerus.^14^,^22^,^28^ This approach requires reflection of the extensor mechanism, typically through either a triceps-splitting approach or an olecranon osteotomy. The transolecranon exposure for distal humerus fractures is a very popular technique that is suggested for improving articular visualization and allowing accurate reduction. Significant osteotomy complications have prompted recommendations for alternative exposure techniques. Distally, intra-articular exposure is dependent on triceps mobilization, and there are many modifications in the posterior elbow surgical approaches: e.g., triceps-splitting, triceps-reflecting, triceps-reflecting anconeus pedicle (TRAP), anconeus flap transolecranon (AFT), and paratricipital approaches.^14^,^22^

**Figure 2:**
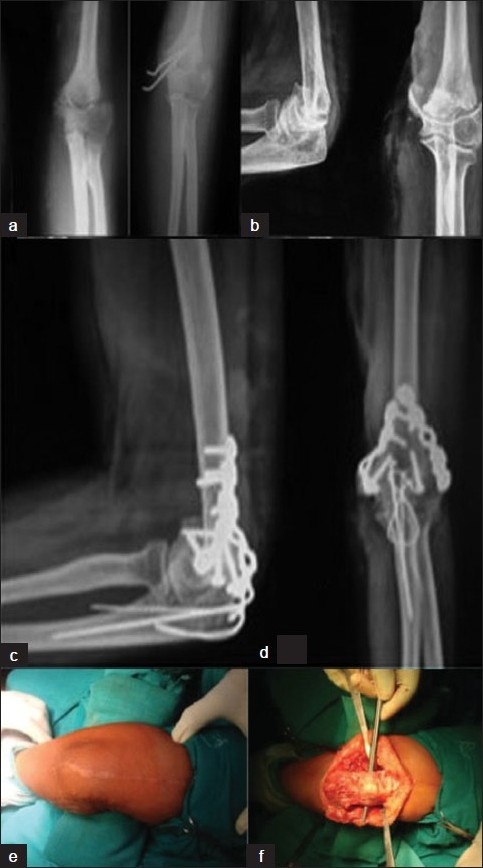
Plain X-ray anteroposterior view (a) shows intraarticular fracture of distal humerus that was treated by K-wire fixation (b); developed nonunion (c); nonunion stabilized by bicolumnar fixation with reconstruction plates and extra-articular olecrano-osteotomy stabilized by tension band wiring (d). Removal of implant was planned by paratricipital incision (e); the first picture shows the postoperative scar; the second picture (f) shows a paratricipital incision for removal of implant.

**Figure 3:**
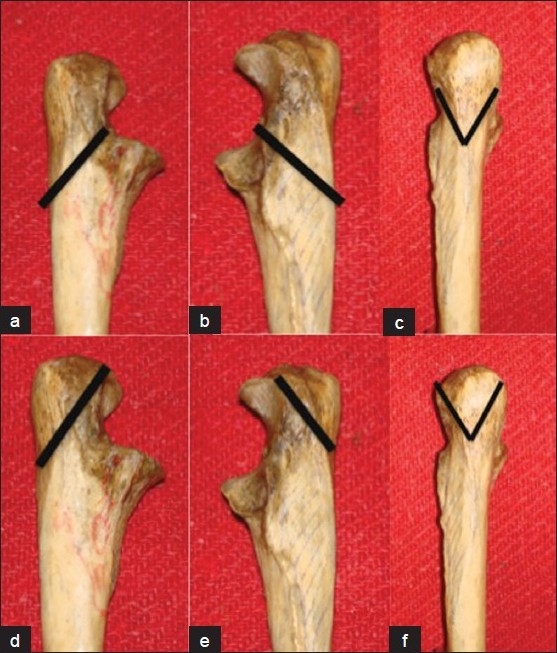
Bone models showing line marking. (a-c) Intrarticular chevron olecranon osteotomy, (d-f) Extraarticular olecranon osteotomy

**Figure 4:**
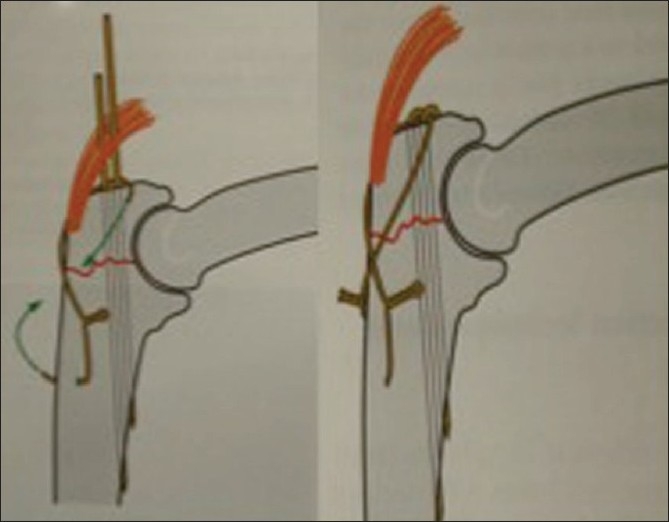
Fixation of Chevron olecranon osteotomy with tension band wiring

**Figure 5:**
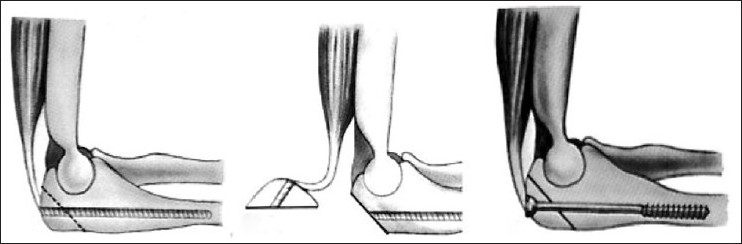
Olecranon osteotomy and fixation by cancellous screw

a) *Triceps-splitting (Campbell)*^27^: A longitudinal straight or curvilinear incision is made from the proximal triceps muscle to the distal triceps tendon, across its insertion on to the tip of the proximal olecranon. After the posterior skin incision is made, the ulnar nerve is identified and protected. Then full-thickness flaps are created medially and laterally by splitting the triceps muscle distally along the central tendon up to the olecranon. The elbow joint is exposed as the anconeus is reflected subperiosteally and laterally off the proximal ulna, and the muscle belly of flexor carpi ulnaris is reflected medially off the proximal ulna. At the end of the procedure the triceps tendon is repaired with nonabsorbable suture. This approach does not provide proper exposure of the distal articular surface.

b) *Triceps-reflecting (Bryan-Morrey)*^17^: In this procedure, the extensor mechanism comprising the triceps tendon, forearm fascia, and periosteum are reflected as one unit from the medial to lateral off the olecranon. The ulnar nerve is first identified and protected. A periosteal elevator is used to dissect the triceps muscle from the posterior humeral cortex. With a scalpel, the forearm fascia, periosteum, and triceps tendon are reflected directly off the olecranon from medial to lateral as a continuous sleeve. The triceps may be removed along with a thin wafer of bone to facilitate bone-to-bone rather than tendon-to-bone healing at the triceps insertion site. Now the entire triceps muscle with the posterior capsule is reflected upwards and laterally, and the elbow is flexed to expose the joint. At the end of the procedure the triceps tendon is reinserted back on to the olecranon by means of nonabsorbable sutures passed through transosseous drill holes in the olecranon. The triceps repair needs protection for 4–6 weeks postoperatively, and the patient should avoid active elbow extension against resistance.

c) *Triceps-anconeus reflecting pedicle (TRAP) (O’Driscoll)*^18^: This approach, where the triceps is reflected in continuity with the anconeus, was popularized by O’Driscoll.^18^ A superficial midline or curvilinear posterior incision is made. The deep exposure involves combined lateral and medial sides of the elbow as per modified Kocher and Bryan-Morrey approach. It usually begins laterally by preserving the lateral collateral and annular ligament, where the anconeus is elevated subperiosteally from the proximal ulna, which is separated from the capsule of the elbow. The anconeus is first exposed distally; the exposure is developed proximally and the muscle is reflected upwards by developing the interval between extensor carpi ulnaris and the anconeus. The common extensor origin and the origins of the extensor carpi radialis longus and brachioradialis are left undisturbed on the humerus. The anconeus tendon and muscle is then separated from the annular ligament and the lateral collateral ligament complex, which are preserved, and dissection continued proximally beneath the triceps muscle. At the level of the tip of the olecranon, an arthrotomy is made and the capsule opened. The medial exposure consists of the triceps-reflecting approach of Bryan-Morrey. An incision is made at the subcutaneous border of the proximal ulna and extended up proximally to the tip of olecranon and along the flexor carpi ulnaris origin on the humerus. The periosteum is then elevated off the subcutaneous surface of the ulna from medial to lateral, continuing beneath the anconeus muscle. At the medial side of the olecranon, the incision is extended proximally along the medial border of the triceps, while retracting and protecting the ulnar nerve. Now the entire triceps muscle with the posterior capsule is reflected upwards and laterally and elbow is flexed to expose the joint. It is important to reattach the triceps precisely at that point after the completion of the procedure, so that the proper length–tension relationship is restored and the mechanical advantage of the olecranon is not lost.

d) *The anconeus flap transolecranon (AFT) approach*^29^: This is a straightforward approach with limited complications. The AFT approach, a combination of the TRAP approach and the olecranon osteotomy, was developed to combine the anconeus preservation of the TRAP approach with the exposure obtained from an olecranon osteotomy. It utilizes an internervous plane to preserve the anconeus muscle and a chevron-shaped osteotomy for maximal joint exposure. After the isolation and protection of the ulnar nerve, lateral portion of the exposure of TRAP approach, the anconeus pedicle is raised proximally through the interval of the extensor carpi ulnaris and the anconeus with its intact nerve supply in a triangular fashion with its base located close to the lateral border of olecranon till the level of planned osteotomy. Subsequently, an apex distal chevron osteotomy is planned that enters the elbow joint at the shallowest part of the trochlear notch. The anconeus muscle flap and olecranon fragment are then elevated off the posterior humerus, exposing the fracture of the distal humerus completely. After repair or reconstruction of the distal humerus, the osteotomy is reduced and internally fixed.

e) *Paratricipital*^30^: The paratricipital approach has the advantage of maintaining the triceps insertion undisturbed and eliminating the risk of postoperative triceps insufficiency. This approach is commonly used for irreparable distal humerus fractures in elderly patients for whom a TEA may be planned in future. The approach is also useful for ORIF of distal humerus fractures, especially transcondylar and supracondylar fractures. Visualization may be compromised by the presence of the intact triceps unit over the elbow joint. A posterior skin incision is made and the ulnar nerve is identified and protected. Medially, the tissue plane between the medial intermuscular septum and the medial side of the olecranon and triceps tendon is developed. Laterally, the plane between the lateral intermuscular septum and the anconeus muscle, which is in continuity with the lateral aspect of the triceps, is developed. The dissection between the medial and lateral tissue planes meets at the posterior humeral cortex as the triceps muscle is released from the humerus. The triceps tendon may be retracted medially or laterally, and fracture fixation is performed by retracting the triceps unit in either direction [Figure 2]. Placing the elbow in an extended position relaxes the triceps and may result in improved visualization of the posterior elbow.

f) *Olecranon osteotomy transolecranon approach*^5^,^11^,^12^,^21^–^24^: For the treatment of intra-articular fractures, a posterior transolecranon exposure by olecranon osteotomy affords optimal visualization of the distal humerus articular surface and both columns of the distal humerus. The olecranon osteotomy could be either extra-articular or intra-articular, both of which expose the distal articular surfaces properly [Figure 3]. A transverse intra-articular osteotomy is inherently unstable and can be difficult to reposition accurately.^16^ In contrast, a chevron-shaped osteotomy, particularly one that has been cracked at the articular surface of the olecranon, facilitates repositioning and has inherent rotational and translational stability due to interlocking of the fragments [Figure 4].^12^,^24^,^26^ Commonly, the osteotomy is created with an oscillating saw in a chevron configuration, typically with the apex pointed distally. An osteotome is used to complete the procedure so that a portion of the osteotomy site is serrated. This serrated area will provide improved interdigitation of the fragments for fixation at the end of the procedure. Extrarticular osteotomy is performed obliquely with oscilating saw exiting close to the tip of olecranon without entry into articular surface [Figure 3]. Several methods of osteotomy fixation are described in the literature: tension band wiring, intramedullary screw fixation, and compression plating. Commonly the osteotomy is fixed with two K-wires [Figure 4] or by using a 4-mm/6.5-mm cannulated screw and washer with an 18-gauge tension band wire [Figure 5].

The drill hole for placement of the wire is located away from the olecranon osteotomy at a distance equal to that from the osteotomy site to the tip of the olecranon. The osteotomy should be repaired by advancing the K-wires into the anterior ulnar cortex distal to the coronoid process.^12^,^24^ After the K-wires have reached the anterior cortex, they are backed out approximately 5 mm and bent 180°, and then tapped back until they are buried under the triceps tendon. The triceps may then be sutured over the wires to discourage backout.^17^ Wire prominence may be more common than actual wire migration, with skin problems and infection.^22^,^23^ The prominence of the hardware can be limited by using two smaller (22-gauge) wires, passing the tension wires beneath the triceps insertion, and bending the Kirschner wires 180° and impacting them into the olecranon.^12^,^25^,^26^ Wire migration may be more likely if the Kirschner wires are drilled down the intramedullary canal of the ulna (where they may not gain strong purchase) and if they are then bent and left above the triceps. Techniques such as drilling the wires obliquely so that they engage the anterior ulnar cortex and then impacting the proximal ends into the olecranon may help limit wire migration. A recent biomechanical study suggested that this orientation of the wires might also enhance the mechanical strength of the construct.^12^

### Surgical technique: Fracture exposure and fixation

It is generally accepted that the transolecranon posterior approach is the gold standard for optimal exposure of intra-articular distal humerus fractures.^5^,^6^,^8^,^14^,^17^,^18^,^21^–^23^,^27^,^29^,^30^ A posterior midline curvilinear incision is made avoiding the tip of olecraon and convexity towards lateral side of elbow so as to avoid chances of ulnar nerve adherence to the scar. Ulnar nerve can be transposed anteriorly if required. The Ulnar nerve is identified medial to the humerus and proximal to the zone of injury, and it is carefully exposed. The cubital tunnel is released and the nerve is freed for anterior transposition if required or planned. An intrarticular chevron-shaped (apex distal) osteotomy is made in the olecranon or, alternatively, an extrarticular osteotomy; this is the preferred method for complete articular exposure of the distal humerus fracture. The olecranon is then reflected proximally by dividing the joint capsule. The triceps is split loosely, medially and laterally parallel to the humerus, taking care to protect the ulnar nerve medially and the radial nerve laterally. The triceps is then lifted off the posterior surface of the bone.

The distal humerus articular surface is reassembled and the intra-articular components of the fracture are reduced first and then temporarily fixed using Kirschner wires. Once the joint surface has been restored and reconstructed anatomically, the metaphyseo-diaphyseal fragments are reduced and again temporarily fixed with K-wires, stabilizing both the columns. The metaphyseal butterfly, if present, is also anatomically reduced to maintain the length of column.^10^ When there is intercondylar comminution or bone loss/bone missing from the trochlea, care should be taken not to narrow the trochlea. This may require placement of a piece of structural graft, usually obtained from the iliac crest. Once satisfactory anatomical reconstruction of the distal humerus has been achieved, a plan can be proposed for final fixation of the distal humerus. There are two ways of fixation of fracture, according to the plan worked out with the help of the paper tracings.

**Figure 6:**
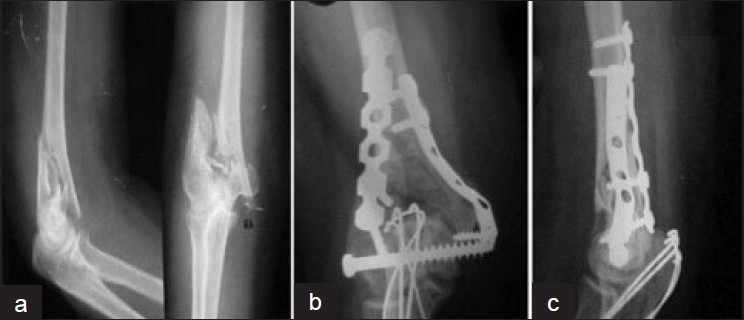
Radiograph (a) showing C2 type fracture stabilized by intercondylar cancellous screw and bicolumnar orthogonal fixation (b) by contoured plates. Extra-articular osteotomy (c) is stabilized by two parallel K-wires and tension band wire

**Figure 7 (a, b):**
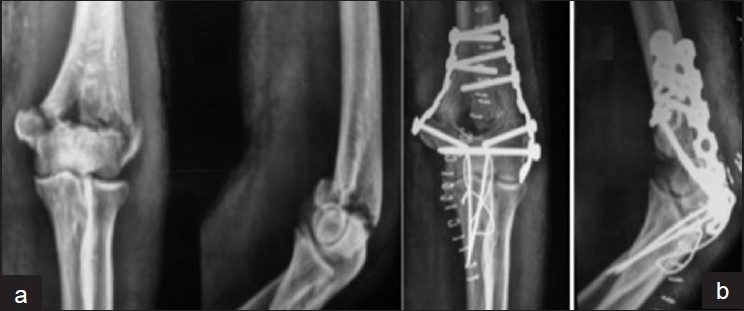
Radiograph showing C2 type fracture stabilized by, intercondyler cancellous screw and parallel plate fixation by contoured plates. Extraarticular osteotomy stabilsed by two parallel K-wires and tension band wire

a) *Type A: 90-90 fixation – orthogonal*^5^,^10^,^21^,^23^: Intercondylar fixation is carried out first using a lag screw, except in the case of a C3 type of fracture, which can be fixed simply by an intercondylar screw. The intercondylar fixation is done either by using a 4-mm cancellous screw or with a malleolar or 6.5-mm cancellous screw, which converts T-Y fracture into a supracondylar fracture. Subsequently, column fixation is done using plates: the lateral column is fixed by a posterolateral reconstruction plate and, medially, another plate is fixed, either reconstruction or Dynamic compression plate.^5^,^23^ On both sides the plate requires precontouring [Figure 6].

b) *Type B: Parallel plate fixation*^2^,^15^,^16^,^18^,^29^–^31^ [Figure 7]: Here the intercondylar lag screw is not used. Instead the fracture is fixed by two parallel plates (medially and laterally) with screws that interdigitate with each other from both sides giving the effect of a fixed-angle structure. Plate length is chosen according to the proximal extension of the fracture line and each plate is fixed with at least three bicortical screws at the diaphysis. The plates are fixed to the distal fragment with cortical screws that extend into the opposite condyle; the proximal fixation is initially done with a cortical screw. Then the fracture is compressed at the supracondylar level with the insertion of an eccentric screw through one of the proximal holes in both plates. An attempt is made to hold the distal fragments together using at least two screws extending to the opposite column. Proximal fragments are fixed according to the configuration of the fracture by at least three bicortical screws. The stability achieved by this fixation construct combines the features and stability of an arch, while locking the two columns of the distal part of the humerus together.^2^,^16^ The concept follows the architectural principles of an arch, in which two columns are anchored at their base to the shaft of the humerus and are linked together at the bottom (long screws from the plates on each side interdigitating within the articular segment).^16^

The medial plate is placed on the medial aspect of the medial column and the lateral plate is placed laterally, rather than posteriorly, on the lateral column. To avoid the stress riser effect, one of the plates should be longer proximally.^2^,^16^ Although the plates are referred to as being parallel, each plate is actually rotated posteriorly slightly out of the sagittal plane such that the angle between them is often in the range of 150°–160°. This orientation permits the insertion of at least four long screws completely through the distal fragments from one side to the other.^2^,^15^,^16^ These screws interdigitate, thereby creating a fixed-angle structure and greatly increasing the stability of the construct. Contact between screws is intended to enhance the locking together of the two columns. The plates must be contoured to fit the geometry of the distal part of the humerus. If precontoured plates are not available, locking plates may be used. Interfragmentary compression is obtained between articular fragments as well as at the metaphyseal level through the use of large bone clamps that provide compression during the insertion of the screws attaching the articular segment to the shaft. In the distal fragments, fully threaded screws inserted in this manner provide maximum thread purchase. The fracture fixation is tested on the table by manually moving the elbow joint and confirming the stability achieved by surgery. The absolute stability allows early range of motion, which is one of the more important advantages of stable rigid fixation. Finally the osteotomy is fixed either by tension band wires over two parallel K-wires or by cancellous screw or by olecranon plate.

### Fracture fixation

Of five biomechanical studies of distal humeral fracture fixation in the literature,^32^,^33^ only three have compared the 90-90 plate fixation (medial and posterolateral plates perpendicular to each other) to parallel plate fixation (medial and lateral plates in the sagittal plane).^34^–^37^ Of these three studies, two showed parallel plate fixation to be substantially more stable than orthogonal plate fixation,^34^,^35^ while one demonstrated no difference.[36] Schemitsch *et al*.^34^ reported the biomechanical superiority of parallel plate fixation (a lateral plate combined with a medial reconstruction plate, applied exactly as described in the report), compared with medial and posterolateral reconstruction plates placed in the traditional manner according to the AO/ASIF (Arbeitsgemeinschaft fur osteosynthesefragen / Association for the study of internal fixation) technique (orthogonal). Parallel plate fixation was found to be substantially more stable than 90-90 plates in all directions tested. Significant controversy exists about whether orthogonal or parallel plating is superior for fixation of distal humerus fractures. Arnander *et al*.,^38^ using a similar model, demonstrated significantly higher strength and stiffness in the parallel group *vs* the orthogonal group when subjected to sagittal bending forces; they also demonstrated a greater stability in compression and external rotation of parallel plating *vs* orthogonal plating. Korner *et al*.^33^ compared standard and locking plates in dual dorsal or orthogonal configuration and found that the torsional stiffness and restraint to sagittal bending were best with orthogonal locking plates. In a few of the studies, the parallel plating technique was shown to be superior to the orthogonal plating technique.^34^,^35^ This system of parallel plating allows greater perioperative stability than orthogonal fixation of 90-90 by using reconstruction or 1/3 tubular plates, as confirmed by the absence of nonunion in the series of Atalar *et al*.^31^ In a study done in 1994 Schemitsch *et al*.^34^ conducted biomechanical evaluation of different methods of internal fixation of the distal humerus. Five different constructs were studied:

1. Two columns posteriorly fixed by plates
2. Single posterior ‘Y’ plate
3. Two plates applied: one posteriorly on the lateral column and the other medially on the medial column orthogonal (90-90)
4. Two plates applied at right angles: posteromedially on the medial column and laterally on the lateral column
5. Two plates applied opposite to each other (parallel-180°), laterally over the lateral column and medially over the medial column

The study concluded that two plates applied opposite to each other – a lateral buttress plate and a medial reconstruction plate (parallel) – achieved maximum rigidity in the absence of cortical contact. Biomechanical and clinical studies have shown that the double-plate technique, where the plates are placed at right angles to each other (orthogonal, medial, and posterolateral), cannot sometimes provide adequate stability for some types of fractures.^7^,^32^,^34^,^35^ To overcome this problem, a parallel plating technique has already been developed by molding the plates to the anatomical curve of the distal humerus.^2^,^15^,^16^ The stability achieved by this fixation construct combines the features and stability of an arch, while locking the two columns of the distal part of the humerus together. In summary, presently, it appears that there is no clear biomechanical superiority of one dual plate configuration over the other.^38^,^39^,^40^

### Ulnar nerve transposition

There is still the controversy regarding whether anterior transposition of the ulnar nerve should be performed routinely at the time of fracture fixation. Ulnar nerve transposition at the time of surgery offers no benefit and in fact may place the patient at a greater risk of neuritis.^43^ Theoretically, transposition has several advantages: it moves the nerve away from the implants, prevents kinking of an incompletely released nerve, and protects the nerve from entrapment by operative scar.^41^ The additional handling and devascularization of the nerve during transposition may result in nerve irritation and this may be one of the reasons for the high prevalence of ulnar nerve symptoms in those undergoing transposition of the nerve.^39^ Hence, a few authors do not recommend routine transposition of the ulnar nerve.^41^,^42^ Some authors advise that the nerve be returned to its normal course at the end of the operation but stress that its position must be clearly recorded so that it can be protected during any later procedure.^13^ Till today, there is no consensus regarding whether ulnar nerve transposition at the time of fracture fixation provides any benefit.^41^,^42^

### Role of primary total elbow arthroplasty

Gambirasio *et al*.^3^ evaluated the functional outcome of ten older patients treated with primary Total Elbow Arthroplasty for comminuted intra-articular distal humerus fractures. All patients were women who had suffered a simple fall but had significant comminution and osteopenia. Although there are many reports of partial or total elbow arthroplasty as a reconstructive procedure for nonunited fractures of the distal humerus, there are few reports of prosthetic replacement of the elbow as the primary treatment for fractures of the distal humerus.^43^ The largest series reported to date of the use of TEA as the primary treatment for distal humeral fractures is by Cobb and Morrey,^44^ where 20 consecutive patients with 21 fractures were reviewed retrospectively. The results of TEA for complex fractures of the distal humerus in this selected group of elderly patients are good. In younger patients the results of open reduction with stable internal fixation allowing early movement are good and TEA is not an acceptable alternative. Joint arthroplasty for displaced fractures of the neck of the femur and head of the humerus in the elderly is accepted practice and hence primary elbow replacement in similar circumstances should be considered. TEA can be a better option than ORIF in the elderly patient with a distal humerus fracture and severe articular comminution in the setting of osteoporotic bone. Cobb and Morrey^44^ reported 100% good or excellent results by the Mayo Elbow Performance Score (MEPS) at a mean of 3.3 years in a cohort of 20 patients treated with TEA for distal humerus fractures. They concluded that TEA is a reasonable treatment for comminuted intra-articular distal humerus fractures in the elderly patient. Disadvantages of TEA include activity restrictions (typically a lifting limit of 5 lb) and the risks of prosthetic loosening, polyethylene wear of the bushing, periprosthetic fracture, and infection. Several studies have compared TEA with ORIF for intra-articular distal humerus fractures. A comparison of case series that studied TEA or ORIF separately found no strong evidence in favor of either.^24^ A retrospective case series of women aged >65 years with distal humerus fractures and associated comorbidities showed more excellent or good results by the MEPS in TEA *vs* open reduction and fixation.^4^

A comminuted distal humerus fracture in an older patient is a difficult clinical problem. ORIF carries the risks of nonunion, loss of fixation, infection, and stiffness. Though TEA carries the risks of loosening, infection, and periprosthetic fracture, it has the advantage that it allows immediate use of the limb and permits elbow function, unlike internal fixation.^3^,^4^,^44^ Both procedures are technically challenging, and complications following these procedures are frequent. Transcondylar fractures of the distal humerus are rare injuries that are caused by low-energy trauma in elderly osteopenic patients. These injuries are characterized by a transverse fracture at the level of olecranon and the coronoid fossae. The small size of the distal fragments has very little bone which is covered with articular fragments; this makes internal fixation difficult and hence primary elbow replacement is the treatment of choice.^3^,^4^

### Rehabilitation

Introduction of an early rehabilitation program along with emphasis on early use of the elbow will improve the functional success of the fracture fixation technique. Gentle active-assisted and passive motion is started early, within the first few days, and routine use of continuous passive motion machine is advisable. The patient can be instructed to support the wrist with the opposite hand and gently flex and extend the elbow, gradually increasing the range of motion.

## CONCLUSION

Adhering to well-described principles during ORIF can help optimize outcomes and minimize complications. The posterior transolecranon approach with anterior transposition of the ulnar nerve gives excellent exposure of the distal articular surface of the humerus. With currently available technology, notably precontoured periarticular locking plates, most displaced distal humerus fractures can be treated successfully with surgical intervention. Although the configuration of dual plating is a matter of surgeon preference, stable fixation of both columns and of the articular surface is required for success. The olecranon osteotomy is preferably fixed by two parallel K-wires passed obliquely into the proximal ulnar anterior cortex below the coronoid and fixed by tension band wiring, with two tightening loops and bend wires that are pushed until they are buried under the triceps tendon. Primary total arthroplasty of the elbow has its own distinct role in the treatment of distal humerus fractures in the elderly patient with severe articular comminution and bone loss, although the proper indications must be recognized.
